# Away from Home or Returned Home? What Iranian Participants with Dementia Experience while Living in a Culturally Profiled Nursing Home in Sweden

**DOI:** 10.1007/s10823-023-09490-6

**Published:** 2023-11-23

**Authors:** Mahin Kiwi

**Affiliations:** Department of Health, Medicine and Caring Sciences, Linköpings University, 601 74 Norrköping, Sweden

**Keywords:** Culturally profiled nursing home, Dementia, Home, Iranian immigrants, Qualitative research

## Abstract

This study explores Iranian immigrants with dementia living in a culturally profiled Swedish nursing home and what it means to be at “home.” The meaning given to a place, in general, is understood to be generated and formed by experiences, expectations, hopes, and chains of events, and its significance can change over time. Life changes will mainly affect the understanding of what constitutes “home.” Such a concept can be challenging to define, especially amongst some immigrant populations and those with dementia, for whom parameters change and choice can be limited. This qualitative research study is based on ethnographic fieldwork following ten participants. The data was analyzed, and three main categories that contribute to understanding “home” as part of the delivery of care to elderly immigrants with dementia, namely “a place to escape to”, “a place to be”, and “a place to live”, were identified. The findings show that living in a culturally profiled nursing home in Sweden gave residents a feeling of self-rule, although the institution did have its codes and rules. Considering the feeling of home, none of the participants felt at home; instead, they stated that the culturally profiled nursing home was merely a place to live. Factors that strengthened their independence were the ability to speak the language they were familiar with and receiving medical help without relying on their children.

## Introduction

To understand the concept of "home", we first may need to know what a place and place-making are, and understanding a place can affect our perception since it has many meanings depending on the individual and the circumstances.

According to Relph (1976, p49)," place is center of action and intention", and the author writes:" To be inside a place is to belong to it and to identify with it." In comparison, Rowles (2017) emphasizes that place is physical and psychological and highlights that human beings' reaction to a place or even the shaping of the place essentially has to do with the person's accumulated experience of life.

Rowles &Watkins (2003, p 3) argue that life is a continuous process of transforming places into meaningful ones through human habitation and habituation. Habitation plays a vital role in shaping one's personal narrative and connection to a specific location, which is how a space can be gradually transformed into a place through social interaction and developing a sense of belonging. However, Rowles et al (2003) accentuate the pivotal role of personal encounters, engagements, familiarity with established patterns, and social connections in shaping one's perception of a place. In alignment with Larsen's insights from 2009, it becomes evident that our understanding and encounters within a specific location dictate our potential for engagement and initiative and leave an indelible imprint on our sense of self. Rowles (2013, p 11) states that space is essential, but place matters more because it expresses the essence of life.

### Place Making

Rowles et al. (2003) discuss the idea of place-making and identify four essential elements: history, habit, heart, and hearth. They emphasize preserving historical significance through tangible objects like photographs or artifacts, which contribute to our sense of identity and connection to our personal and historical experiences. The authors also highlight the importance of habituation and daily routines in creating a meaningful place, as it shapes our personal story and connection to a specific location. The idea of home is seen as a personal space where people feel comfortable and find their identity. It is vital for emotional well-being and is connected to the need for ownership and belonging. The authors emphasized the significance of home as a central place in our lives. It is a place of safety and security and holds special meaning for some people. "For many people, the most intense expression of being in place is provided by the concept of" home" and the experiences of being at home." (Rowles, 2013, p 11).

Already ten years previously, Rowles & Watkins stated:" We suggest that being in place results from the repeated and cumulative process of making spaces into places." Having a place mixed with our personal pleasant and satisfying experiences makes the place feel" home". (2003, p 79)

For some immigrants, moving to a residential care facility may be the last move in a long chain of abandoned homes in various countries – sometimes having left under circumstances forcing them to do so rather than out of free choice (Kiwi et al., 2018). For these people, the sense of home and their actual home have been redefined repeatedly.

Although this is the case for a growing number of people because of global migration, little research has been conducted on how immigrants with dementia define and experience their “home.” As Madison (2006) notes, migration – mainly when made from choice – raises existential severe questions about inter alia the understanding of oneself, belonging, freedom, independence, loss, and home. He suggests that home may best be defined as the place where the individual and the environment “match” (2006, p 12) and that it may be understood better as a ‘person environment, *interaction*’ rather than as a “place.” Again, little research has been conducted about how this affects self-understanding, perceptions of their needs and the best way to help them feel at “home” as individuals and in the community.

### Defining What " Home " Means

Defining what " home " means has not been an easy undertaking. Although the term is very familiar and central to our daily life, a general understanding revolves around and is strongly linked to safety and freedom. Zingmark et al. (1995) defined it as recognition, control, initiative, freedom, safety, privacy, and togetherness. In comparison, Oswald et al. (2006) identified the meaning of home, among others, as ‘emotional’, i.e., a bond involving the experiences of privacy, safety, pleasure, and stimulation. The authors emphasize that the individual's meaning of home is the result of the place-attachment process. Rowles and Chaudhury (2006, p 82) noted, ‘Attachment to a place for elders and how such attachment gets intimately linked to a preservation of a sense of personal identity’. Furthermore, home is the ‘fulcrum’ of the world for elders, and they ‘cognitively differentiate the physical environment into zones of decreasing intensity of involvement away from their homes’, i.e., emotional attachment’ (Rowles, 1984, p. 143). A similar statement is to be found by Frank (2005), home is a concept connected to human memory and emotion. As such, we all are connected psychologically to the home, where accumulated memories reside.

Possessions also shape and reinforce our sense of self in our home. Their residence is significant for many people as it represents their identity and personality (Rowles et al., 2015, p 319). As well as their identification of the concept of home, Rowles and Chaudhury reveal its somewhat sad – but undeniably true – “face”: ‘[…] the context of the home may provoke a range of emotional experiences […] [it] may evoke ambivalent or negative emotions; for example, when family abuse takes place in the privacy of home’ (2006, p 9). Surprisingly, not much is written about home as an unsafe place.

There are two central and fundamental views of the home. First, it is a place-based concept related to the physical environment, and second, a more abstract perspective sees it as a symbol representing family, belonging, love, security, and personal identity (Frank, 1999, 2005), each of which develops over time.

### The Sense of Home

The sense of home for older adults is considered a vital resource. Despite their different and distinguished lifestyles, Oswald et al. (2006) emphasize that the sense of home becomes more relevant for older adults who spend most of their time at home.

Ahmadi Lewin (2001) emphasizes that “home” for older adult immigrants means an intense change, involving leaving their home country to live in a new one, which can have different physical and psychological impacts. Furthermore, despite the cognitive challenges that Alzheimer’s disease entails, an impression of home remains in the mind (Frank, 2005) beyond a physical place.

Even though the concept is complex, it can be understood on two primary levels: first, as a place-based concept related to the physical environment, and secondly, as a symbol representing family, belonging, love, security, and personal identity (Altman & Werner, 1985; Doyle, 1992; O’Bryant, 1982; Rubinstein, 1989; Sixsmith, 1986; Sixsmith & Sixsmith, 1991). The second level is more abstract and represents a sense of home (Bulos & Chaker, 1995; Frank, 1999), independent of an actual building or structure; instead, it develops in people over time.

### Dementia and the Concept of Home

Despite the cognitive losses that dementia causes, people with dementia can comprehend the concept of home and convey what it means through personal expressions such as drawings and verbal definitions, thereby illustrating the poignancy connected to the term. As mentioned earlier, home is strongly connected with emotions. Therefore, it is crucial to understand the reasoning for someone with dementia to maintain a sense of home through statements such as “I want to go home.” This statement can be understood through three domains: automaticity, universality, and semiotics (Frank, 1999).

### Nursing Home, Autonomy, and Independence

'An older adult's home might be a comforting, familiar place although it is becoming burdensome to maintain and unsafe (and therefore a source of anxiety)' (Rowles & Chaudhury, 2006, pp 23). To what extent one can be viewed as independent and the degree to which one can "reduce" or even "measure" autonomy and independence in a nursing home has not been addressed at any great length in the literature.

McColgan (2005) refers to Savishinsky (1991), regarding how nursing homes are curiously named because they offer little medical cures or much that can be said to be like home. Furthermore, they are usually public places, denying a true feeling of having a private life.

Nursing homes try to do something for their residents by recreating a feeling of home, but through surveillance and schedules, they unintentionally impede their progress in this regard. The nursing home provided for the residents in this study did not feel at home, made evident by their constant attempts to leave. Teatime was a way for many of them to escape their surroundings mentally and have a chance to reflect on home and the past with other residents (McColgan, 2005). A study by Paterniti (2003), gave examples of residents adapting nursing home schedules to meet their wants, for example, by constantly eating later than the assigned times, refusing to shower when told to do so, and attempting to do scheduled activities earlier than usual.

### Culturally Profiled Nursing Home (CPNH)

According to the Swedish statute, a person with dementia should be placed at the center of any care and nursing intervention. For instance, individuals with significant issues may need 24-h care and medication, which can be offered at specialist dementia nursing homes. The National Guidelines recommend that social services should offer extraordinary accommodation/nursing homes adapted explicitly to people with dementia (The National Board of Health and Welfare (NBHW) 2018).

Anahita (the fictive name of the Culturally profiled nursing home states that: Anahita is a Persian facility for those of you who long for home in your elderly days. At Anahita, we need more than just speaking Persian. When you arrive at Anahita, we want to create a sense of being home in Iran'. We decorated with the help of furniture, carpets, smells, paintings, and other things reminding us of the homeland. There are smaller sitting groups for those drinking tea, and there are possibilities to smoke the water pipe. We have all the classic party (parlour) games, Persian TV, Persian radio, and Persian music. Here at Anahita, we show respect and consideration for the different views on gender existing in Persian culture. For older adults, it can be essential to be cared for by someone of the same sex. Family is important. When you move to Anahita, we are foremost a complement to your family. We will not take over. Your family will still be able to have responsibility.

The NBHW defines person-centered care: ‘When proving person-centered care, it is essential to consider the needs of people with a different cultural or linguistic background. Cultural considerations include, for example, giving the person the opportunity to practice their religion, eat culturally adapted foods, preserve, and maintain their cultural traditions, customs and gain access to staff who speak the same mother tongue, have cultural competence, and can provide security, increase well-being and a sense of home for the person with dementia (2018, p. 86).

In the culturally profiled nursing home (Anahita), all residents had their furnished room, a kitchen, a toilet, and a bathroom, and often family members would bring in reminders (family pictures, mats) from their previous homes. In addition to their own space, they would gather in the dining room to eat together, and in the absence of family members or friends, staff members would often be regarded as surrogates. The only item the older adult received from the nursing home was a bed.

Every resident had a member of staff as the contact person. The contact person reported to family members with the necessary, urgent status report or in case the resident required extra favourite hygiene items. If a family member wanted to drive the resident home for some occasion, the contact person was responsible for preparing him/her, packing the resident's bag, and so on. Sometimes communication did not work as both parties wished, but cooperation between them often went on without any challenges. If the resident's contact person were off duty that day, another staff member would assist the resident.

Residents had different kinds of activities. The activities depended on the ability and needs of the residents. Once or twice a week, they had sit gymnastics. Sit gymnastics was very popular, and many residents participated. Otherwise, when the weather was suitable, they went for a walk. There were individuals who, regardless of the weather, enjoyed going for a walk, especially those who were smokers. Once a week, they played bingo and liked it very much; almost everybody won something, such as hygiene items or chocolate. Residents had different kinds of activities. The activities depended on the ability and needs of the residents. Once or twice a week, they had sit- gymnastics. Sit gymnastics was very popular, and many residents participated. Otherwise, when the weather was suitable, they went for a walk. There were individuals who, regardless of the weather, enjoyed going for a walk, especially those who were smokers. Once a week, they played bingo and liked it very much; almost everybody won something, such as hygiene items or chocolate.

Celebrations, birthdays, and Mother’s- and Father’s Day were essential issues. All traditional and national celebrations were also considered to give the residents a feeling of home. The "concerns" and "sorrows" that relatives/family members had in common (their family member with dementia lived in the culturally profiled nursing home) made them come together, and they often sat and talked to each other about the advantages and disadvantages of the nursing home.

### Prayer Room

A prayer room is a cultural consideration. According to NBHW, it is vital to consider needs related to people with different cultural or linguistic backgrounds. Cultural considerations include, for example, allowing the person to practice their religion (NBHW (2018). Most residents in this nursing home were of the Islamic faith, but several Christians were also. The culturally profiled nursing home had organized a prayer room for any resident who wished to perform daily prayers, but this room was never used for its intended purpose. There was no interest in having a prayer room. Not using the prayer room does not necessarily mean that a person lacks spirituality or a sense of meaning in his/her life.

### Feeling at Home in the Nursing Home

Numerous scholars have investigated the factors influencing residents' feelings of being at home in nursing homes. Rijnaard et al. (2016) studied the various factors contributing to a sense of home among older adults. They identified two critical situations that support this process. First, a sense of home is fostered in a secure, comfortable, and predictable environment, with residents Fedel at ease and can genuinely be themselves. Second, they found that certain factors significantly impact this sense of home, including psychological, social, and the built environment within the nursing home.

Robinson et al. (2010) emphasizes the significance of having a sense of home, specifically through relational care. Relational care involves understanding the individual beyond their dementia and facilitating regular conversations between staff and family members. As Van Hoof et al (2015) stated, a person who values ​​independence, stability, the source of one's identity, freedom of choice and control, and memories ultimately establishes a sense of belonging associated with personal experiences and emotions.

Place integration refers to the ongoing process in which individuals engage with specific places in their real-life experiences. It involves the continuous effort people put into improving their relationship with these places, leading to a reconnection with the location and the formation of new significance in their lives. In essence, place integration highlights the meaningful and transformative connections individuals can establish with the places they encounter (Cutchin, 2005).

According to Marden et al. (2001), as dementia progresses, many people move into residential care facilities that often attempt to create a sense of home. Due to the different transitions, they have experienced (migration, coping with the onset of dementia, etc.), it is essential to establish their experiential perspectives and contemporary understanding of what home means. However, life values become rooted in day-to-day interactions, so this study aims to explore what Iranian participants with dementia experience while living in a culturally profiled residential home in Sweden.

## Method

### Setting and Participants

Anahita, the first culturally profiled residential home for Persian-speaking individuals (regardless of religion) with dementia, opened its doors in 2010 in Sweden with a capacity of 20 residents (Table 1). During the fieldwork, three passed away; seven had significant speech difficulties and were non-communicative.

**Table 1:** The number of rooms in each floor and residents at the cultural profiled home (Anahita)

Floor	Floor 1	Floor 2	Floor 3	Floor 4	Floor 5
Language	Arabic	Persian	Persian	Persian	Swedish
The number of rooms in each floor	10	10	10	10	8
Number of Male	3	5	4	2	3
Number of Female	7	5	6	8	5

Dementia ward (Floor 2 and 3)

The criterion for inclusion in this study was that they should be an Iranian immigrant. In addition, they should have already been assessed by the Swedish medical care system and been diagnosed with dementia. Degrees of individual cognitive skills varied, and the participants were recruited based on easy access to the home, i.e., convenience and purposeful sampling (Yin, 2015). Ten participants were selected because they could engage in conversation, and interviews were conducted in Persian, Azerbaijani, or a mixture of languages.

#### Ethical Considerations

The study was approved by the Central Ethical Review Board (EPN) (Rec No: 2012/180–31), in accordance with the Declaration of Helsinki (World Medical Association, 2013). All participants and stakeholders were given verbal and written information, following which they provided written consent. Pseudonyms were applied for all participants.

#### Data Collection

The methodology used herein is ethnographic (Brewer, 2000), and data were collected via interviews, observations, and field notes. As Spradley (1979b) and Skinner (2012) state, the central aim of ethnography is to understand different ways of life, life patterns, and social situations Ethnography is ‘not a matter of method but an intellectual effort’ (Geertz, 1973, p. 2), implying that it goes beyond data collection to involve local experience, whereby ethnographers think like locals and act as outsiders. It also entails asking for and gaining the intimate trust of the studied group. Etic and emic approaches are not isolated, as their combination is a dominant methodological approach. Harris (1976) emphasizes that emic description requires etic and emic as an actual disclosure of the potential interactive context in ethnographers meeting with the participant and discussing something dominant. Emic refers to any conceivable disclosure in such a meeting.

The fieldwork began in mid-November 2012 and continued for a year. Observations were performed on weekdays, weekends, holidays, and evenings. The researcher was a “nonparticipant,” although, at times, staff received the researcher’s assistance whenever it was asked for or required.

During the first visit, the researcher was introduced to all staff levels at the nursing home. While greeting residents, Spradley’s (1979a, b, c) emotional and lyrical expression came to mind for conceptualizing the bond between interviewers and participants: ‘I want to understand the world from your point of view. I want to know what you know in the way that you know it. I want to understand the meaning of your experience, to walk in your shoes, to feel things as you feel them. Will you become my teacher and help me understand?’ (Spradley, 1979b, p. 34). Data was collected via in-depth interviews, i.e., a conversation with a purpose (Silverman, 2013).

The researcher focused on the participants’ versions of their experiences and how these were portrayed. The interviews had to be dementia friendly to gain access to the participants' knowledge.

Often, to help people with dementia remember, a photo may be a straightforward tool. I used photos from family albums and sometimes different pictures to help relax the interviewee. In addition, this method helped introduce new subjects, and the participants described their perceptions, experiences, and understanding related to specific questions, people, times, places, and events. Benninger & Savahll (2017a, b) believe that using photos offers adults and children an alternative form of communication.

Interviewing individuals with dementia produces interesting, surprising, and unexpected answers that can sometimes catch the interviewer off guard. For example, when reminded about the inconvenience of forgetfulness, they would state, "I do not forget – I just do not remember."

Most of the residents had gradually forgotten their second language, and it was a precondition that I could adjust myself to shifts in language during our conversations. Therefore, the interview could be half in Persian and half in Azerbaijani. Furthermore, because of the progressively decreasing communication ability, facial expressions, gestures, touch, eye contact, nearness, and voice volume/tone became more critical in interpreting their mediation (Wimo & Sandman, 1999).

Most of the interviews were conducted in the participant's room, sometimes with a favorite staff member, a relative, or just the two of us. All conversations were recorded and lasted between half an hour and an hour. Conversations were completed at different times since I used to spend a good deal of time with them daily, weekly, and monthly. The interviews were supplemented and constructed during the fieldwork through interaction with the residents as well as observations of the nursing home. These observations were also recorded.

All interviews were recorded, de-identified, and transcribed verbatim in the relevant language. Ages ranged (Table 2) from 67 to 99 years old, and anonymity was applied throughout. The residents I interviewed had lived in CPNH shortly after the nursing home was established.

**Table 2:** Characteristics of participants living with dementia diagnosis (PWD)

Gender	Age	Duration of stay in Sweden	Length of time of diagnoses prior to moving to the residential home
Female	79	15	2 Years
F	90	10	4 Years
F	75	26	1½ months
F	88	20	2 Years
F	87	29	1 Year
F	67	25	2 years
F	84	18	3½ years
F	89	18	5 Years
F	80	18	5 years
Male	93	24	6 months

The interviewed individuals with dementia diagnosis living at the culturally profiled nursing home (Anahita)

#### Data Analysis

Transcripts of the interviews, observations, and field notes were analyzed to locate and classify the meaning of “home.” This study used inductive content analysis and followed the four steps of the content analysis presented by Elo and Kynges (2008), namely repeated readings of interviews and the creation of an overall picture, 2) open coding, i.e. marking phrases and sentences, identifying relevant statements and freely generating codes to categories before listing, 3) creating categories, i.e. grouping into higher-order headings (not only bringing together similar categories but also classifying them according to where they belong according to their content) and 4) abstraction, where general descriptions are formulated. Each category was labeled with some content characteristics (2008).

The data collected for this study, i.e., recorded interviews, texts, and notes, all dated and timed, were translated into available languages ​​and double-checked by secondary individuals. Such reviews or controls were included in the institution's guidelines. A researcher with expertise in qualitative methods validated the analysis.

As the researcher, my language knowledge, and cultural awareness were helpful in many ways, but I was there to reveal their views. I strived to learn my informants' everyday life, experiences, and history as they saw them (especially as I knew Iran before the revolution, and they could remember a lot about that time as well). In addition to Persian, I spoke several other languages ​​used in Iran). I, like many others with my Iranian background, have my own personal experiences from migrating to Sweden as a student. The informants’ family formation, reunification, and life in Iran and Sweden differed for every individual.

## Findings

The participants fled their “homes” (Iran) and ways of life at various times, before the 1978–1979 revolution and during and after the war between Iraq and Iran in 1980–1988.

Every person's life has its patterns and content, regardless of status, traditional way of life or standard of living. Clarity regarding migrating to Sweden and being with their children became evident in the interviews. Family reunification for almost all participants revolved around the desire to live together in harmony and security. Most participants’ health was good before they moved to Sweden and/or were reunited with their children. The most incredible feeling of security was together with family members, so they saw Sweden mainly as a place to escape.

According to the participants, their life situations changed after moving, and despite the reasons for emigrating, or their unique reasoning for doing so in the first place, they saw it as a place to stay.

Despite all the pain and joy that had previously been part of their lives, they had been diagnosed with dementia, which forced them eventually to take up permanent residence in a nursing home, thereby resulting in relocation to a third place – a place, according to the participants, where one was no longer dependent on children's support and practical help. A nursing home let them avoid seeing their children struggling to support them, eliminating what they saw as constant worries about their parents' fragile state of health.

**Fig. 1:**
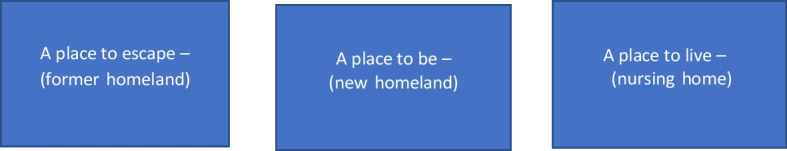
Overview of the three categories

From all the interviews, three leading opinions on Sweden – in contrast to Iran – were established: Sweden had become a place to escape to, a place to be, or a place to live. (Fig. 1).

### A Place to Escape to

There are many reasons why people emigrate to another country, perhaps due to mentally and emotionally pleasant or unpleasant experiences of a home that has been “left behind.” Life histories and memories, i.e., “archaeology and landscapes,” in one way or another remain with a person and may be the starting point for every step taken in life when facing a new reality. “A place to escape to” was based on the participants’ lives in their former homeland, which formed the basis of their rudimentary memories. Some participants, on the one hand, recounted pleasurable experiences, in contrast, for others, the bloody wars, such as with Iraq, and the absence of safety and security led to an escape from fear.

Whether we like it or not, our experiences in life can affect us in one way or another. Especially those living with dementia can often – and in the best way – identify far better what has happened in the past than in the present. Some female participants talked about their home life back home in Iran and health conditions in Iran and Sweden.

In most societies, women have many responsibilities, mostly centering on their roles as mothers and housewives. The character and grade of commitment may differ from one family to another. However, the transformation of this unit, from a traditional structure to another, is highly demanding, as one female participant emphasized:

Hana: I worked at home, and I had far too much to do: kids, house, cooking, laundry, organizing the home and guests, friends, and relatives. I didn’t have much time to be able to work in an office.

Nonetheless, all this responsibility gave her a sense of belonging.

The female participants had lived different lives – some easy and others hard. Some carried the heavy, traditionalist expectation of what was required of a woman or wife, as exemplified by Ayna, who felt degraded and that her humanity had been disregarded:

From the field notes, in an informal interview during a “fika” evening (a traditional Swedish social gathering, typically around coffee and pastries):

Interviewer: What is it that most makes you sad, Ayna?

Ayna: When all others claim that I did not take care of my husband, he humiliated me. Now I cry – not because he’s dead, but over my lost respect and health.

The participants also made statements about the spectre of family abuse, and regardless of whether the violence was verbal, physical, or emotional, it was not uncommon to hear the same types of oppressive stories:

From the field notes, during “fika,” together with Gilda at the nursing home where she lives, talking about her journey to Iran during the summer:

Interviewer: Would your life have been different if you lived in Iran?

Gilda: No, never. I always wanted to escape from an oppressive home environment… I did not have a good childhood; no good memories at all. I was 13-14 years old when my stepmother… married me to a man. He was much older than me and was even a drug user… and [he] used to beat me… Due to constant beatings, I lost my hearing and had a stroke. My husband insulted me and threatened to kill my children and me...

Physical, psychological, and verbal abuse meted out by this 80-year-old’s husband was a regular occurrence. In years gone by, it would be common to marry girls to older, relatively wealthy men, as it would be easier to mould them into obedient wives, thereby depriving the girl of any self-respect.

Another form of verbal abuse would involve Gilda’s husband commenting that other women in the surrounding area were better than her, backed up by the threat that he would marry another woman, thereby forging the perception of her home environment.

In 1980, a long war broke out between Iraq and Iran. After the eight-year conflict ended, Iran adopted an Islamist theocratic approach to government, forcing many of the participants’ family members to leave. According to some participants, the aggressive attitudes shown by Iran towards them gave them a feeling of rejection, in contrast, others felt that they were in Sweden only for the sake of their children, who had fled during the war and then brought their parents to the safety of a new country. A few other participants had stayed in Sweden for various reasons without originally planning to remain.

Longing for the motherland, or “touching the green grass of home,” was something the participants often expressed. When asked whether they would like to be back living in Iran, I received different answers:

Interviewer: What would your life be like if you were still in Iran?

Ayna: I never think of Iran. Iran has never had a place in my heart. In Iran, there is no brotherly feeling of being in harmony with or being friends with others.

Interviewer: If you were in Iran, how would your life be different from the one you live here?

Nina: The life that we all once had will never return. What joys me now is that my children are here –and I´m here for my children´s sake.

As human beings, the participants were the result of their experiences, memories, perceptions, and accumulated historical capital, all of which influence the strength of belonging to their homeland and immediate environment.

What the key factors of security and safety meant to the participants was not always obvious, but all of them agreed it was a necessary part of their lives and involved several aspects besides political security and safety:

Nina: Iran is not a country for us to live in. I was born in Tehran and grew up there, but Teheran was once more safe and secure…

They stated that war had destroyed their lives, first through revolution and then between Iraq and Iran:

Diba: We had a good life in Iran... I had a wonderful life until the first revolution, and then the war destroyed everything for me and my family…

Home can be considered the best refuge for a person, depending on their values and experiences. Regardless of gender or age, some participants described their past lives through a sense of longing:

Arta: There was nothing that we lacked. It was not just me but everyone who lived in Iran. But after the revolution ... [tailed off and did not want to discuss it].

### A Place to be

According to the participants, migration to Sweden was often based on their children’s desires, as they thought their parents should not be back home alone in Iran in their old age. Many participants emigrated for medical treatment, to be with their children or relatives, and/or to create or develop a harmonious environment in which to live. In most cases, they developed an emotional attachment to their new country of residence.

Family reunification, for most of the participants, was an ideal solution in that it allowed them to come together and leave various problems and loneliness behind, albeit it was not without its issues:

Ayna: My son… left me and married… He does not think about me at all… I get despondent [she cries]… I am a mother; I raised a child not to abandon me, or at least to ask how I am or how my life is.

A peaceful and harmonious home environment is the desire of most people. However, but it did not necessarily happen for some participants, who found themselves thrust into an environment where their children often argued – and seldom agreed – with them. In the culturally profiled nursing home, they found a place away from these conflicts, and although it was not easy to achieve, the place gave them a sense of contentment:

Aida: These conflicts between my children affected my health a lot. They never understood that I, as a mother, had had a heart attack and surgery in Iran. None of my kids took my situation into account.

Almost all of the participants’ children had left Iran due to war, and either at the beginning of the war or later, they reunited as a family. However, it was not always the parents’ solitude that raised concerns among their children in Sweden about a family reunion since almost all participants who moved to Sweden already had a connection to the country. Some initially had no plans to stay:

Gilda: My daughter is the reason we ended up in Sweden. My daughter had problems with her ex-husband, who was Iranian. We were there to help her and the children. I took nothing from my apartment because we had no plans to stay… But somehow, we did stay…

Some had stronger intentions not to move away from their home country but wished to remain in their new country because of their family situation. Their children’s different problems in Sweden had, in one way or another acted as driving factors for family unification:

Diba: Despite everything, I lived in Iran, but I was told that my son here in Sweden had MS, and he needed my help. I therefore came and stayed here without any plans to stay.

Being reunited with family does not mean ending solitude or basic needs. Almost all of the participants originally had their apartments. They lived with their spouse(s) or alone, while others attended Swedish language courses to establish themselves in society or were active in associations and cultural activities:

Ayna: I went on a course to learn Swedish, and I even learned painting from a lady… I do not know where she comes from, but we painted together.

The feeling of loneliness was also linked back to the participants’ former homeland. In Iran, friendships were established earlier in life, but they had disappeared one by one, partly due to war or to migration.

Losing a life partner, for many, accelerated the journey to total loneliness or isolation. Their shared daily routines shared happiness and sadness were gone, and psychological, physical and social loneliness took over, as exemplified by Nina:

Nina: I was alone, even though sometimes my daughter would visit... Nevertheless what is sad is my beloved husband’s death… We lived for 60 years together. Sixty years is a person’s life.

For some participants, the grief was not about loneliness but rather the circumstances of their home life in Sweden. In one case, they had not given their mother any information about the apartment where she had lived with her beloved husband or the possessions from her home. Withholding information made her feel profoundly wounded and sad:

Ida: My husband became ill, and the disease took him away from me, and I was very much alone. After my husband’s death, I became ill and had no information about what had happened to my apartment and the life I had together with my husband here in town. My child did not inform me and never talked about it. It hurt me badly.

The feeling of being deprived of a specific role, losing control of her home, and facing exclusion by not being informed by her children, pained her deeply. The accommodation in which she had lived, regardless of any comfort, was a place to be and something to treasure dearly. Instead, the nursing home became her place to be, as it was comfortable and secure. However, for some, the absence of a life companion resulted in loneliness and subsequent health problems.

Nina: I had a comfortable life here in Sweden. My husband died, and I became lonely. Gradually I became depressed. My children used to come to me and sleep at my house, but they could not always continue to do that…

Even though the participants grew to accept that the nursing home was a place to be, they nevertheless lamented the loss of their previous life:

Diba: I worked hard to build a life – and I succeeded – but suddenly the ‘tsunami of my life destiny’ destroyed everything… The life I had built up with the hope of living with my husband and my children became a dream, an illusion, and a mirage.

Dementia, as well as residential homes, were not familiar concepts to the participants. Most mentioned and expressed their health problems in various ways, such as the aforementioned ‘*tsunami of my life destiny*’*.* Not living together with her husband and children had tormented her, and so her transition from a healthy life to a life with dementia completely transformed both her life situation and the places to be.

The main argument for family reunification was that elderly family members should not remain alone at home in Iran. However, according to the participants, the desire to care for an elderly family member became problematic, thereby decreasing the family caregiver’s ability to provide such care at home:

Field note, during “fika,” together with Vida:

Vida: After I became sick, my daughter moved me here because her apartment was not big, but her husband – and even her daughter – was very nasty. They did not want me to live there with my daughter.

Some of the participants had lived with their children since moving to Sweden, but taking responsibility for a loved elderly family member was not always easy, especially if working full time, and this occasionally gave rise to making a difficult choice:

Arta: I could no longer stay with my daughter.

The expectation of help from children may be evident to many, but there are also negative consequences, such as causing tension among family members and leading to unhappiness and loneliness:

Sara: My daughter’s family problem hurts me badly. I am very lonely and feel abandoned.

As a result of the above issues, the culturally profiled nursing home within this study was perceived as a place to live.

## A Place to Live

After finally moving to a culturally profiled nursing home, the participants realized that relinquishing their home was not a big problem and that being independent of their children was a relief. However, without their children, they still needed some assistance. The nursing home provided personal feelings of safety, security, and independence, which was satisfactory. The personal meaning of feeling cared for by some staff was positive.

Being able to trust the staff also engendered a sense of security in the participants, most of whom suffered from decreased mobility, vision, or hearing, and so, besides ambient assistance and support, walkers, and wheelchairs had become a necessity:

Interviewer: What is it that is not good here?

Ayna: I have seen everything good here; everyone helps, and they are lovely. These days, it is not easy to get help from anyone, even if it is a person's child.

In addition to having access to medical services at any time, the children were no longer worried, which increased the feeling of security:

Nina: I am grateful. Here I get the security that I need to have. My children are no longer worried; if something happens to me here, there are doctors, nurses and others who will help me immediately.

In the culturally profiled nursing home, the participants gathered around the table for breakfast, lunch, and dinner. They also enjoyed different social activities, such as bingo and fitness classes, which helped reduce isolation and loneliness:

Interviewer: How is it going with your neighbours?

Ida: We are three people who meet every day in the cafeteria. They each, like me, have a room. However, my children know her family – and that is good in its way. We sit in the living room, listen to the radio, and sometimes watch TV.

Seeing others with the same lifestyle and background living in the same nursing home gave the participants a sense of joy. Physical proximity can contribute to positive social relationships:

Ida: Some had as good a life as I had in Iran, and today we are here together, and we will see how far fate and destiny will take us...

Being independent is complex and can have different meanings, depending on the relationship between the receiving party and the party supplying help and support. Some people’s lives, for instance, are interdependent, meaning they inevitably depend on each other. The participants’ statements about depending on help from others may be associated with feelings of worthlessness, loneliness, and powerlessness:

Ayna: I am grateful to the Swedish government, which has given me a life. … [It] has quenched my thirst. Now, besides God’s help, I don’t need any help from my children.

Although some of the participants did not speak openly about their feelings around autonomy, they said that becoming dependent on their children was not a good outcome:

Arta: When one gets older, one should not live with one’s children. I am happy I came here because I do not feel like I am a burden and need of my children’s help.

Many people wish to avoid dependency and strive to be independent despite their different physical weaknesses or mental and economic status. Individual needs regarding food management, hygiene, and the like sometimes make them dependent on relatives’ help and support, which can feel stressful, belittling, and burdensome. The reason that the participants’ former homes gave them pleasure was that they were not dependent on their children:

Aida: Believe me, I ask God at least ten times daily to call me home… to him. Nevertheless, I am happy that I am not living with my children.

Some individuals felt that if the nursing home did not exist, they would have been kicked out of their children’s homes:

Guldmay: I am very happy. I can say more than delighted… If I had not gotten this place, my daughter… would have thrown me out, and I would be sleeping out on the street.

Some people were grateful that they received extra services all the time:

Guldmay: I have never opened my refrigerator door to take out some food or fruit. These people here always help me, and I’m incredibly grateful that I live here and not on the street.

Pleasant feelings were evoked among the participants when staff spoke a common language, as well as the presence of a Persian-speaking doctor, making the nursing home a place to live:

Arta: All these are Iranians – the nurses and the doctor speaks Persian. We have professional staff who take care of us. Doctors prescribe the medication that I need.

The participants did not need to stand in a queue or wait for days to see a doctor, and so they felt their needs were met:

Diba: When I need a doctor's help, I get it immediately… They do everything for me.

The participants lived in the culturally profiled nursing home because of dementia, and often they expressed their appreciation in this regard. One participant said that she was happy, but only for her children’s sake, as they had to decide to transfer her to the nursing home due to work and life commitments:

Nina: I like it here, as my children are happy. They did not have much time, and they were worried about me. My daughter said many times that she had trouble concentrating at work… Now they are happy that I am here, and I am happy, too.

Iranian food was essential to some of the participants, as they connected it to their nationality:

Diba: The food is Iranian, and all of us here are origin Iranians.

One participant associated Swedish food with her stay in the hospital and thus expressed dissatisfaction:

Ida: Oh no, God will protect me. When one talks about Swedish food, I think of hospitals [all laugh]. I have been in the hospital several times and am tired of it.

Almost everyone felt comfortable about the fact that the common language was Persian or Farsi, as it increased their sense of security and well-being:

Ida: The language is good, and we all speak Persian.

The importance of speaking a common language was emphasized particularly as relating to a place they could call “home:”

Nina: It is crucial. I could not stay where I could not speak their language, even if they offered me a daily interpreter.

Some people find inner motivation, so it is vital to know in what way(s) they find strength in the “gloominess.” Privacy was the most essential aspect for the participants. All people need to socialize, be recognized, be appreciated, and be a part of a group. There is also a need to be separate and distinct from others, to be a person, and to have one’s private domain.

A resident prayed in her room, but according to her daughter, she did not know what she was saying. She had never previously prayed but would use this as an excuse to go to her room.

Even if she was unfamiliar with the precise prayers owing to dementia or not, she likely adopted this practice for other reasons, such as finding comfort and solace in solitude.

Some of the participants underlined the importance of meaningfulness and security through the freedoms of privacy, spirituality, and meditation, though no one used the available prayer room in the nursing home:

Interviewer: When you sit alone, you talk to God, you told me once. Do you pray according to the Islamic tradition?

Aida: Never. Why should I do it? But I talk with my God. I meditate in my own way, especially on Fridays. I sit and talk to my God and say I have lived my life so far...

Regarding praying in the Islamic way, it is essential to know that prayer consists of verses in the Arabic language, and residents living with dementia used to have difficulties remembering their second languages.

There was also a resident who became unhappy when talking about prayer and the prayer room, without saying why, but after a few weeks the resident's grandson told me: “Grandma lost all six of her sons in prison in Iran, my uncles were executed.”

The female resident who grew sad and depressed when discussing the prayer room may have had a profound emotional connection to prayer because of the tragic death of her six sons in Iran. When addressing prayer, she may be overwhelmed by the pain and anguish involved with such an occurrence.

People have unique ways of connecting with their spirituality, and they may prefer a different form of spiritual expression through meditation and poetry, like the verses from Omar Khayyam.

A Christian resident had a portrait of Jesus crucified and an image of Mother Mary with Jesus in her arms on the wall of his room but did not go to the prayer room. The person meditated by reading the following poem by Omar Khayyam aloud: “Who is the man below who has never sinned, tell me? He who had never committed one, how could he have lived, tell me? If, because I do evil, you punish me with evil, what difference is there between you and me, tell me?" (The English translation in FitzGerald (2010)).

Spirituality and purpose may be found through various outlets, and activities other than traditional religious rituals.

The discretion to influence and create their inner peace through privacy, meditation, and a simple conversation with their God was also significant:

Interviewer: I see you have your rosary and meditate frequently.

Gilda: Yes, I speak with my God; I often speak with him and to him.

Finally, some participants found the nursing home a great place to live. They and their families knew they were safe and receiving reliable care, and they no longer felt burdensome to their children and received a sense of independence. They were also taken care of according to their preferences, which engendered thoughts of home, i.e., being in a place to live.

## Discussion

This study aimed to explore what Iranian residents with dementia experience while living in a culturally profiled nursing home in Sweden.

The findings highlighted three leading categories: “a place to escape to,” “a place to be,” and “a place to live.”

Sweden’s commitment to placing individuals with dementia at the centre of any care intervention means 24-h attention, for instance, can be offered at specialist dementia nursing homes NBHW (2018). According to the National Board of Health and Welfare’s care model, it is vital to consider needs related to different cultural or linguistic backgrounds, including giving the person the opportunity to practice their religion, eat culturally adapted foods, and have access to staff who speak the same language, thus giving a sense of being at home.

Contrary to the more traditional Iranian family care-based system (Abdollahpour et al., 2012; Hajighasemi, 1994), the Swedish model is run by the welfare state and is based upon the philosophical aim of providing necessary support to live an independent, high-quality life. Before the 1990s, most of this care was provided by institutions. However, for financial and philosophical reasons, staying as long as possible in one’s own home is generally considered beneficial –otherwise known as the ‘stay-at-home’ policy. The prevailing opinion is therefore, that, for most individuals, it is better to age in a place with proper support (such as home help etc.) and avoid – as far as possible – relocation to a residential home (Söderberg et al., 2013).

Residential care is a diminishing, if not uncommon phenomenon; for instance, in 2013, 12.9% of those aged 80 + lived in a residential home, compared to 30% in 1975 (Fukushima et al., 2010; NBHW 2018).

Many participants moved to reunite with their children (Hajighasemi, 1994), while others escaped war, hence “a place to escape to.”

According to Hull (1979), one should try to understand the relevant standards and codes of one’s new country when acclimatizing into a society. After settling in Sweden, many participants eventually became psychologically fragile and dependent on help, brought on by loneliness and grief. Some received help from home care services and family, but after years of supporting their parents, their children stopped being family caregivers, so they had to move to a nursing home (Kiwi et al., 2018).

They compared their past lives – with or without being oppressed and abused – to their lives of self-respect and no harm.

As Frank (2005) noted, home can also be seen as a concept connected to human memory, emotion, and positive and negative feelings. Our memories are incomplete, so our extreme highs and lows are what we connect with at home (Willcocks et al., 1987). Despite the specific cognitive issues that Alzheimer’s causes, a sense of home appear to remain, and sufferers can comprehend this concept beyond a physical place and convey verbally and through drawings what it means to them (Frank, 1999).

Regardless of living with dementia or not, almost all attributes we ascribe to home are based on our distinct memories, as the environment we live in may remind us of unforgettable events in our lives. Bassett and Graham (2007), refer to Zerubavel (1997) that the social environment plays a significant role in defining what is memorable and what is forgettable.

What the participants could often recall were childhood memories, many of which were sad, while others described their former lives with longing and that not having family members around them meant that they had no feeling or sense of home.

The loss of life companions and friendships was an underlying factor in making the participants feel lonely in Sweden. Emami et al. (2000) highlighted difficulties for elderly Iranian immigrants living in Sweden because of significant differences between the two cultures, and their lack of Swedish language proficiency. However, Klein (2001) in her study of Iranians in Sweden, writes: “They are much more Swedish than we are”, which is also a frequent comment from Swedes who have visited relatives or friends in the United States (2001, p. 67).” According to Klein (2001), some Iranian families who visit those in Sweden claim that Iranians here are real Swedes (their food culture and social culture are not the same as Iranian ones), and most do not follow Iranian national traditions.

Another painful issue for some participants is what gerontologists call ‘role reversal’ (Emami et al., 2000), i.e., children nurturing their parents. Despite this conflict, however, family cohesion was still strong, as demonstrated when deciding to move parents to a nursing home so that they could be cared for appropriately (Kiwi et al., 2018). Life in Sweden, and in the nursing home, made most participants feel respected and safe because they received attention and regular, targeted help.

In addition, some thanked the Swedish government and society for not having to depend on their children, thereby giving them a sense of autonomy, self-respect, and strength.

Even though the concept of home is in many ways complex, it can be understood, as Altman and Werner (1985) state, on two primary levels: the physical environment and belonging, love, security, and personal identity.

As well as physiological and cognitive reasons for being in a nursing home, underlying factors include a sense of belonging, acceptance, respect, and safety. Solem (1996) emphasized that these feelings are neither right nor wrong; instead, they are true.

Often what the participants experience as security, safety, and respect may depend on how the CPNH creates a sense of home for them, i.e., as McColgan, (2005) states that Mandela (2002) noted, we build our home through our experience and thoughts.

Security came in the form of trusting staff, access to medical help, speaking a common language, familiar food, etc., thereby agreeing with what Oswald et al. (2006) and Tryselius et al. (2018) state, namely an emotional bond involving the experiences of privacy, safety, pleasure, stimulation, and a setting existing only as an attribute and as an emotional environment.

In Rowles and Chaudhury’s (2006, p 82) notion that ‘Attachment to a place for elders and how such attachment gets intimately linked to a preservation of a sense of personal identity’, it was noted that home is the ‘fulcrum’ of the world for elders and that they ‘cognitively differentiate the physical environment into zones of decreasing intensity of involvement away from their homes’, i.e. emotional attachment (Rowles, 1984, p. 143).

This attachment is also confirmed by Nygaard et al. (2020), who contended that subjective feelings direct attachment to a place and give a sense of home, which can result in familiarity and attachment to physical places and people.

In a study on the views of residents with dementia living in nursing homes, participants talked about daily activities, social gatherings, and feeling safe and secure. The concept of home was very emotional and awakened many associations (Nygaard et al., 2020), similar to the present study.

Many of the Iranian immigrants came from different sociocultural backgrounds. Regardless of previous status or life conditions, they had now reached a new “Heimlich”, i.e., familiarity and homeliness, because of the sense of security and privacy (Heimlich was an early psychological concept of the symbolic home formulated by Freud (1919), as they were no longer dependent on their children and feeling of greater security was provided. By being able to speak their native language and having a familiar diet, they went through a kind of repatriation transition, as suggested by Sussman (2000), without having to move back physically to their own country, which was no longer how they remembered it.

The participants had issues of feeling like they belonged in Iran, but simultaneously they noted and admired the Swedes’ humanity. They were, therefore, not as estranged in Sweden as they would have been by staying in Iran. As Madison (2006) notes, sometimes migration brings a person’s internal sense of self closer to their external environment than they felt in their country of origin. Thus, a sense of belonging can be essential in naming a home.

In order to understand the concept of "home," it is essential to understand what a place is.

Rowles and Bernard (2013) discuss the concept of place, place-making, and identity. They stress the importance of preserving historical significance through tangible objects like photos or artifacts, which contribute to our sense of identity and connection to our personal and historical experiences. The authors also highlight the significance of daily routines and habits in creating a meaningful place, as they shape our personal story and connection to a specific location. The idea of home is viewed as a personal space where individuals feel comfortable and find their identity. It is crucial for emotional well-being and is linked to the need for ownership and belonging. The authors emphasize the central role of home in our lives, as it provides a sense of safety and security and holds special meaning for many individuals. Overall, our understanding and experiences within a specific location shape our engagement and sense of self. Rowles and Bernard emphasize that while space is important, place holds a greater significance as it expresses the essence of life. This understanding of home and space is in conformance with the present study's findings.

Several studies have examined the factors contributing to residents feeling at home in nursing homes, not specific nursing homes for dementia. Rijnaard et al. (2016) found that a secure and comfortable environment and various psychological, social, and built environment factors play a significant role in fostering a sense of home. 

Robinson et al. (2010) highlights the importance of relational care, which involves understanding the individual beyond their dementia and facilitating conversations with staff and family members. Van Hoof et al. (2015, 2016) emphasize that a sense of home is associated with personal experiences and emotions, including independence, stability, and freedom of choice. According to Cutchin (2005), place integration is an ongoing process in which individuals engage with specific places, forming meaningful connections.

Marden et al. (2001) argue that as dementia progresses, residential care facilities attempt to create a sense of home, but it is essential to understand individuals' experiential perspectives. The present study aims to explore the experiences of Iranian participants with dementia living in a culturally profiled residential home in Sweden. The residents who lived in CPNH did not mention that it was their home. They just saw it as a place to live.

A place to live is impacted by variables like being together with people from the same socio-economical background: living in CPNH together with people from the same economic background may create a sense of familiarity, which may promote the feeling of comfortable and may ease the adjusting to surroundings in the CPNH.

Admittance to medical services: availability of medicine, care requirements, visiting doctors and nurses at any time, and having positive experiences, may contribute to more comfort in CPNH. Not wanting to burden their children and live an independent life in CPNH was occurring expressions from the residents.

The importance of a common language lies in effective communication, social cohesion, and cultural exchange. Common language promotes a feeling of belonging to the Community. Common language may reduce misunderstandings, and ease communication and interaction. Almost all residents had problems with the Swedish language before they began to live with dementia. Communication with the doctor in another language rather than your own may give rise to different challenges. According to Swedish law, every individual with a language than Swedish has the right to get the help of an authorized interpreter.

When they wanted to visit a doctor or other authority they communicated by an interpreter, however, using everyday language gave them a sense of ease in CPNH.

The amount of social interaction a resident receives varies from individual to individual and day to day. Sometimes no one spoke to each other. Sometimes they held each other warmly and walked up and down the corridor. Moreover, other times they would admire each other and introduce the person to his/her family. Partly it was because their relatives would greet someone, which affected the resident’s attitude toward the person the family greeted. Not all interactions were peaceful, though (Kiwi, 2018).

Feeling safe and secure can be one of the vital facts for individual health and well-being. Some residents have had unpleasant experiences in the home they were living in before. Being neglected, socially isolated, or losing independence threatens feeling safe. Living in a CPNH can give the residents this safety.

Because of various factors, the food has had significant importance and represents the traditional culinary heritage. Food gave a sense of contentment and connected them to an often sense of satisfaction, if not permanently.

Some of the residents' experience of satisfaction in a CPNH depended on the staff's pleasant attitudes, showing kindness, and helping them by giving them service, which gave them a feeling of well-being. Kindness toward them created comfort, trust, value, and respect and reduced fear and exclusion. However, stigmatization and judgment by the staff sometimes made family members and friends worried that they would not be taken care of properly (Kiwi, 2019). Generally, personal satisfaction was experienced within activities both outdoors and indoors, celebrations of birthdays, other kinds of special days, national days, Iranians, the Swedish New Year's Eve, and social gatherings.

### Limitations and Strengths of the Study

This study was based on ethnographic data following ten participants only, but despite this low number, there was a good amount of rich data. Another possible limitation is that only one Iranian culturally profiled nursing home was studied so that future research could include similar nursing homes in other Scandinavian countries. The strength of the study is that there was no need for an interpreter, so conversations flowed freely and with focus. Furthermore, although the participants’ descriptions may have been subjective, these personal experiences drove this study and its findings.

## Conclusions

Living in a culturally profiled nursing home in Sweden meant taking the feeling of home into account; individual personal experiences of home played a significant role, and although none of the participants felt at home, all of them stated that the culturally profiled nursing home was a place to live. Factors that strengthen Iranian immigrant participants’ independence are speaking their familiar language, receiving medical help without relying on their children, and feeling safe and secure. It is crucial to consider the needs of people with different cultural or linguistic backgrounds. Cultural considerations include, for example, allowing the person to practice their religion, eat culturally adapted foods, and preserve and maintain their cultural traditions and customs if this is what the person wants.

## Data Availability

Data are available in Persian, a mix of Persian, and Azerbaijani, and translated into the Swedish language. Data is not available to outsiders due to ethical and privacy reasons.

## Appendix

### Interview Guide (Dementia Friendly, Guiding the Participants Through a Conversation)

Tell me about yourself (in this photo).

What did you do in Iran? Did you work at home? If you were back in Iran, what would your life be like?

Do you miss Iran? Would you like to go back there? What makes you homesick?

Tell me about your life in Sweden – what is best? Why did you come to Sweden? Did you come after or before your children? How many of them are in Sweden? Did you live with them and, if so, did you like living with them?

Did you work, study or become a housewife when you moved here?

Why did you choose this (place/nursing home) as you say home?

Do you like it in the home? Do you have friends here? Did you eat the same food in Iran? Do you go out to visit your children?
